# Production of ethanol and arabitol by *Debaryomyces nepalensis*: influence of process parameters

**DOI:** 10.1186/2191-0855-3-23

**Published:** 2013-05-09

**Authors:** Himabindu Kumdam, Shweta Narayana Murthy, Sathyanarayana N Gummadi

**Affiliations:** 1 Applied Industrial Microbiology Laboratory, Department of Biotechnology, Indian Institute of Technology Madras, Chennai 600 036, India

**Keywords:** *D. nepalensis*, Arabitol, Ethanol, Fermentation, Bioreactor, Optimization

## Abstract

*Debaryomyces nepalensis*, osmotolerant yeast isolated from rotten apple, is known to utilize both hexoses and pentoses and produce industrially important metabolites like ethanol, xylitol and arabitol. In the present study, the effect of different growth substrates, trace elements, nitrogen concentration and initial pH on growth and formation of ethanol and arabitol were examined. Optimum conditions for maximizing the product yields were established: glucose as carbon source, an initial pH of 6.0, 6 g/L of ammonium sulphate and addition of micronutrients. Under these best suited conditions, a concentration of 11g/L of arabitol and 19 g/L of ethanol was obtained in shake flask fermentations. The fermentation was scaled up to 2.5 L bioreactor and the influence of aeration, agitation and initial substrate concentration was also determined. Under optimal conditions (150 g/L glucose, 400 rpm and 0.5 vvm) ethanol concentration reached 52 g/L, which corresponds to a yield of 0.34 g/g and volumetric productivity of 0.28 g/L/h, whereas arabitol production reached a maximum of 14 g/L with a yield and volumetric productivity of 0.1 g/g and 0.07 g/L/h respectively.

## Introduction

Lignocellulosic biomass represents the largest source of renewable energy, which is primarily composed of cellulose, hemicellulose and lignin. It forms the basis for the production of various value-added products by microbial conversion (Saha 2003; Kumar and Gummadi 2011; Kumar et al. 2008). For bioconversion, pre-treatment of lignocelluloses is important and the resulting hydrolysate contains high amount of hexoses and pentoses (Moiser et al. 2005; Saha 2003; Saha et al. 2005). Hemicelluloses are heteropolysaccharides composed of short chains of sugar units such as xylose, arabinose, mannose, glucose, galactose and glucuronic acids which can be acetylated or methylated (Chandel et al. 2010; Saha 2003; Saha et al. 2005).

Ethanol production by microbial fermentation has drawn considerable attention in the recent years because of its increased use as a fuel, often blended with gasoline. Lignocellulosic biomass has been considered a promising substrate for the production of bio ethanol (Balat and Balat 2009; Sanchez and Cardona 2008). One of the major impediments in utilizing lignocellulosic biomass as a feedstock is the inability of many yeasts to utilize both the hexose and pentose sugars, which are the principal components of hydrolysates from lignocellulose (Yuan et al. 2011; Tian et al. 2009; Sanchez and Cardona 2008). The ability of yeasts to metabolize these hexoses and pentoses has been studied widely. Yeast strains such as *Candida tropicalis, Pichia stipitis* and *Pachysolen tannophilus* were reported to ferment glucose/xylose mixtures to ethanol and xylitol (Hahn-Hägerdal et al. 2007; Sanchez et al. 2002; Sanchez et al. 2008). *Candida entomeae* and *Pichia guillermondii* were known to produce xylitol and arabitol from hydrolysates containing xylose and arabinose (Saha and Bothast 1996). Saha et al. (2007) showed that *Zygosaccharomyces rouxii* NRRL 27624 is capable of producing arabitol and ethanol from glucose but could not metabolize arabinose, which also is a prime component of hemicelluloses.

Arabitol, a pentitol that exists in both D and L forms, is sweet, colourless, crystalline and soluble in water with potential applications in food and pharmaceutical industries. Currently the production of arabitol is by the chemical reduction of lactones of arabinonic and lyxonic acids. This process requires an expensive catalyst and a very high temperature of 100°C. D-arabitol is used for synthesis of compounds which are enantiopure and have medical properties fit to be used as immunosuppressive glycolipids and herbicides (Koganti 2012). Arabitol plays an important role in energy controlled diet, as it is absorbed slowly by the digestive system. It is referred to as anti-cariogenic since it cannot be easily metabolized by the oral flora. Hence it has also been used as a common ingredient in chewing gums (Groleau et al. 1995; Koganti 2012).

*Debaryomyces nepalensis* NCYC 3413*,* a halotolerant strain that has been previously isolated from rotten apple, is capable of utilizing both hexoses and pentoses; and is also important in production of commercially valuable products such as arabitol, xylitol and ethanol (Gummadi and Kumar 2006a; Kumar and Gummadi 2011; Kumdam et al. 2012). In this study, the metabolism of *D. nepalensis* using glucose as the carbon source has been studied under different physiological conditions in both shake flask and bioreactor.

## Materials and methods

### Maintenance medium and inoculum preparation

*Debaryomyces nepalensis* NCYC 3413 was maintained on a solid YEPP medium containing yeast extract 10 g/l, peptone 20 g/l and pectin 5 g/l at pH 7.0 and incubated at 30°C for 26 h and later stored at 4°C. A single colony was transferred from an overnight-grown culture plate into the YEPD medium (50 ml) containing yeast extract 10 g/l, peptone 20 g/l and dextrose 20 g/l and incubated for 12 h at 30°C and 180 rpm.

### Media composition and culture conditions

Semi-synthetic medium containing carbon source, 100 g/l; (NH_4_)_2_SO_4_, 3 g/l; MgSO_4_, 0.1 g/l; K_2_HPO_4_, 6 g/l; Na_2_HPO_4_, 3 g/l; yeast extract, 1 g/l; CaCl_2_·2H_2_O, 147 mg/l; FeCl_3_, 10 mg/l; MnSO_4_·H_2_O, 3.38 mg/l; ZnSO_4_·7H_2_O, 8 mg/l; CuSO_4_·5H_2_O, 0.5 mg/l; citric acid, 6.9 mg/l. The initial pH of the medium was adjusted to 7.0 using H_3_PO_4_ and NaOH. All the components are autoclaved separately and mixed subsequently so that the final medium volume was 50 ml in 250 ml Erlenmeyer flask. The influence of environmental and nutritional factors on growth and product formation was studied by varying the nutrient composition and physical parameters (pH and oxygen availability), which will be mentioned at appropriate section of the manuscript.

#### Cultivation in shake flasks

Shake flask experiments were carried out in 250 ml conical flasks with a working volume of 50 ml. The medium was inoculated with 2% (v/v) of inoculum and incubated at 30°C & 180 rpm for 120 h. Samples were collected at regular intervals to measure growth and the concentration of metabolites. All the samples were centrifuged at 8,000 rpm for 5 minutes. The supernatant was used for estimation of important metabolites (ethanol, arabitol and glycerol) and substrate utilization by high-performance liquid chromatography and the cell pellet was used to quantify growth.

#### Bioreactor studies

Batch fermentations were carried out in a 2.5 L stirred-tank reactor (Minifors, Infors HT, Switzerland) with a working volume of 1.5 L. In all the experiments, the temperature was maintained at 30°C and the initial pH was adjusted to 6.0 and controlled using 2N H_3_PO_4_ and NaOH. The production medium was inoculated with 8% (v/v) of the seed culture and incubated for 6–10 days. The broth was agitated at different speeds (350 to 700 rpm) and the aeration levels were varied (0.3 to 0.5 vvm) depending on the study. Foaming was controlled by adding 2% polypropylene glycol if necessary. Samples were collected periodically and analyzed by HPLC. The dissolved oxygen concentration in the medium was continuously monitored using a DO probe (Mettler Toledo).

### Analytical methods

The growth was evaluated by measuring the optical density of culture at 600 nm (OD_600_). As standardized previously for *D. nepalensis*, absorbance 1.0 at 600 nm corresponds to 0.335 g cell dry weight per litre culture. Samples that were collected at regular intervals were analysed for metabolite production and substrate utilization. The cells were separated from culture medium and the supernatant was stored at 4°C (not more than 24 h) for further analysis. The concentration of glucose and other metabolites arabitol, ethanol and glycerol were estimated by HPLC (Jasco) equipped with refractive index detector and Aminex HPX-87H column (Bio-Rad, Richmond, USA) at 45°C with 0.01N H_2_SO_4_ solution as the mobile phase at a flow rate of 0.6 ml/min as described earlier (Kumar and Gummadi 2011) . The volumetric oxygen transfer coefficient (K_L_a) was determined by the static gassing-out method (Stanbury et al. 1995). The dissolved oxygen (DO) concentration in the fermentor was first brought down to zero by sparging the medium with nitrogen. Later air is sparged at a known flow rate and the increase in dissolved oxygen concentration was monitored with respect to time using a dissolved oxygen probe.

## Results

### Effect of carbon sources

**Figure 1:**
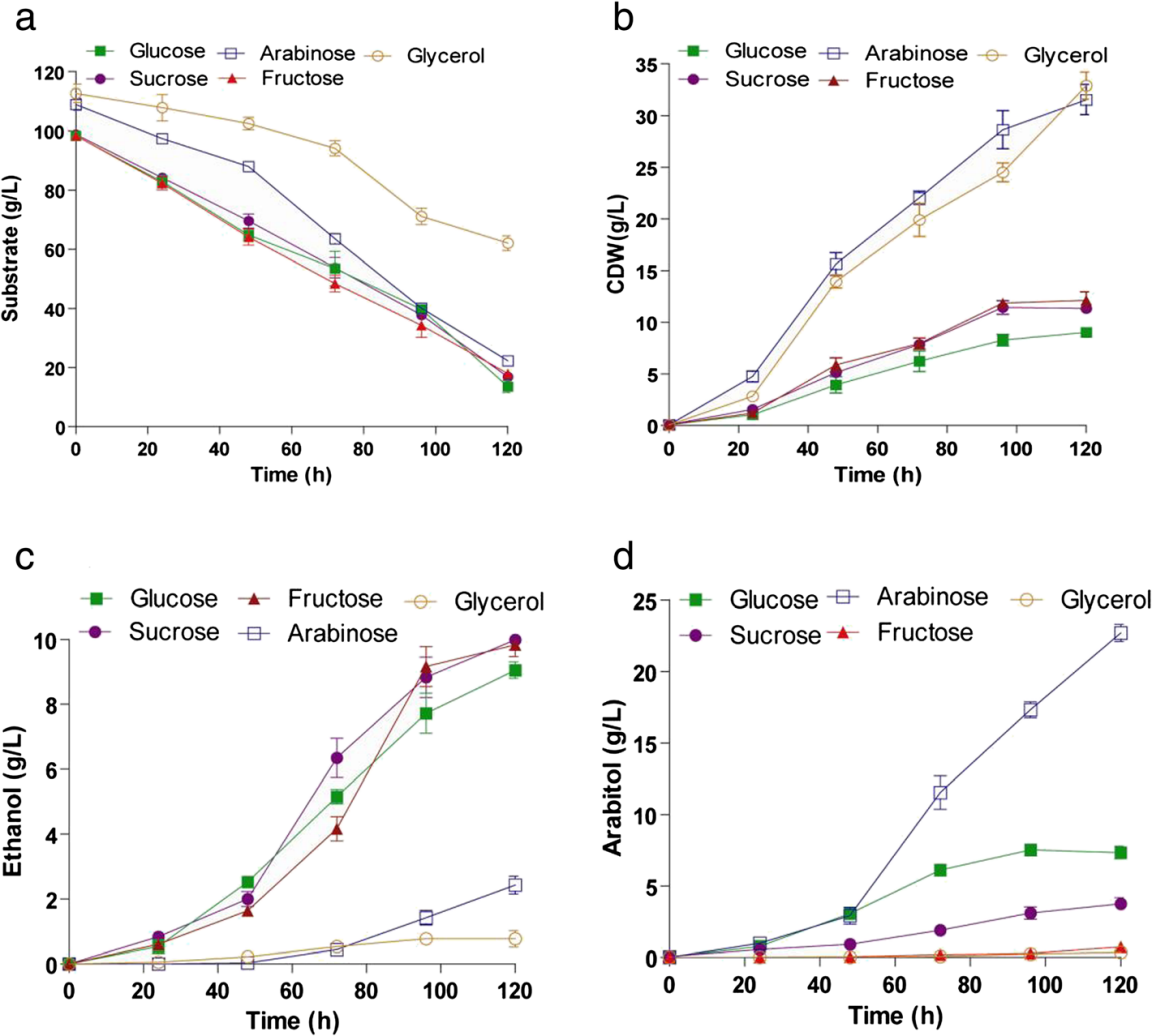
**Production of ethanol and arabitol by *D. nepalensis *NCYC 3413 in the presence of various carbon sources.** Time course of (**a**) substrate utilization, (**b**) growth and formation of (**c**) ethanol and (**d**) arabitol. Fermentation was performed in 250 ml shake flasks with 50 ml of medium supplemented with different carbon substrates and grown at 30°C and 180 rpm for 120 h. All experiments were performed three times and the values reported are mean with standard deviation.

The effect of different carbon sources on growth and metabolite production was determined by supplementing the medium with different growth substrates like glucose, fructose, sucrose, arabinose, glycerol, starch and carboxymethyl cellulose (CMC) such that carbon content was same. The maximum biomass obtained was 33g/L with glycerol and 31 g/L for arabinose, whereas glucose, fructose and sucrose produced 10 g/L biomass (Figure 1b). The substrate utilization profile was almost same for all the substrates except for sucrose, whose consumption rate was low (Figure 1a). Ethanol production was same when glucose, fructose and sucrose were used as carbon sources, where as very low levels of ethanol was obtained with arabinose and glycerol (Figure 1c). The yield of ethanol was 0.12 g/g when fructose and sucrose were used as carbon sources, which is slightly greater than that obtained with glucose (0.1 g/g) (Table 1). Arabinose was found to be best substrate for arabitol production (20 g/L) followed by glucose (5 g/L) (Figure 1d). The organism was unable to produce any metabolite using glycerol and a major portion of the substrate was converted to biomass with a yield coefficient of 0.92 g/g. The organism was unable to metabolize complex carbohydrates like starch and CMC (data not shown). Hence, in further studies, glucose was used as carbon source for concomitant production of ethanol and arabitol.

**Table 1:** Effect of various medium components on the production of ethanol and arabitol

**Parameter**	**Substrate consumed (g/L)**	**Ethanol (g/L)**	**Arabitol (g/L)**	Yield (g/g)*	
**1. Substrate**				**Ethanol**	**Arabitol**
Sucrose	82.00	9.90	3.70	0.12	0.05
Glucose	84.75	9.05	7.30	0.11	0.09
Arabinose	86.70	2.43	22.70	0.03	0.26
Fructose	80.40	9.84	0.70	0.12	0.01
Glycerol	50.60	0.77	0.00	0.02	0.00
**2. pH**					
5.0	51.85	2.95	2.45	0.06	0.05
5.5	57.20	5.00	3.10	0.09	0.05
6.0	91.40	11.50	10.30	0.13	0.11
6.5	84.30	8.96	8.60	0.11	0.10
7.0	84.80	9.50	8.10	0.11	0.10
**3. Level of (NH**_**4**_**)**_**2**_**SO**_**4 **_**(g/L)**					
3	84.70	12.20	9.00	0.14	0.11
4.5	86.50	13.30	8.50	0.15	0.10
6	91.70	18.90	11.60	0.21	0.12
**4. Trace elements**					
Positive control	89.70	19.20	10.30	0.21	0.11
Negative control	59.30	5.70	1.10	0.10	0.02
- ZnSO_4_	95.20	14.80	1.50	0.16	0.02
- FeCl_3_	98.90	12.40	1.80	0.13	0.02
- CuSO_4_	59.40	6.20	1.70	0.10	0.03
- MnSO_4_	54.75	6.20	2.00	0.11	0.04

* Y_P/S_ is the amount of product formed (g) per gram of substrate consumed.

### Effect of initial pH

**Figure 2:**
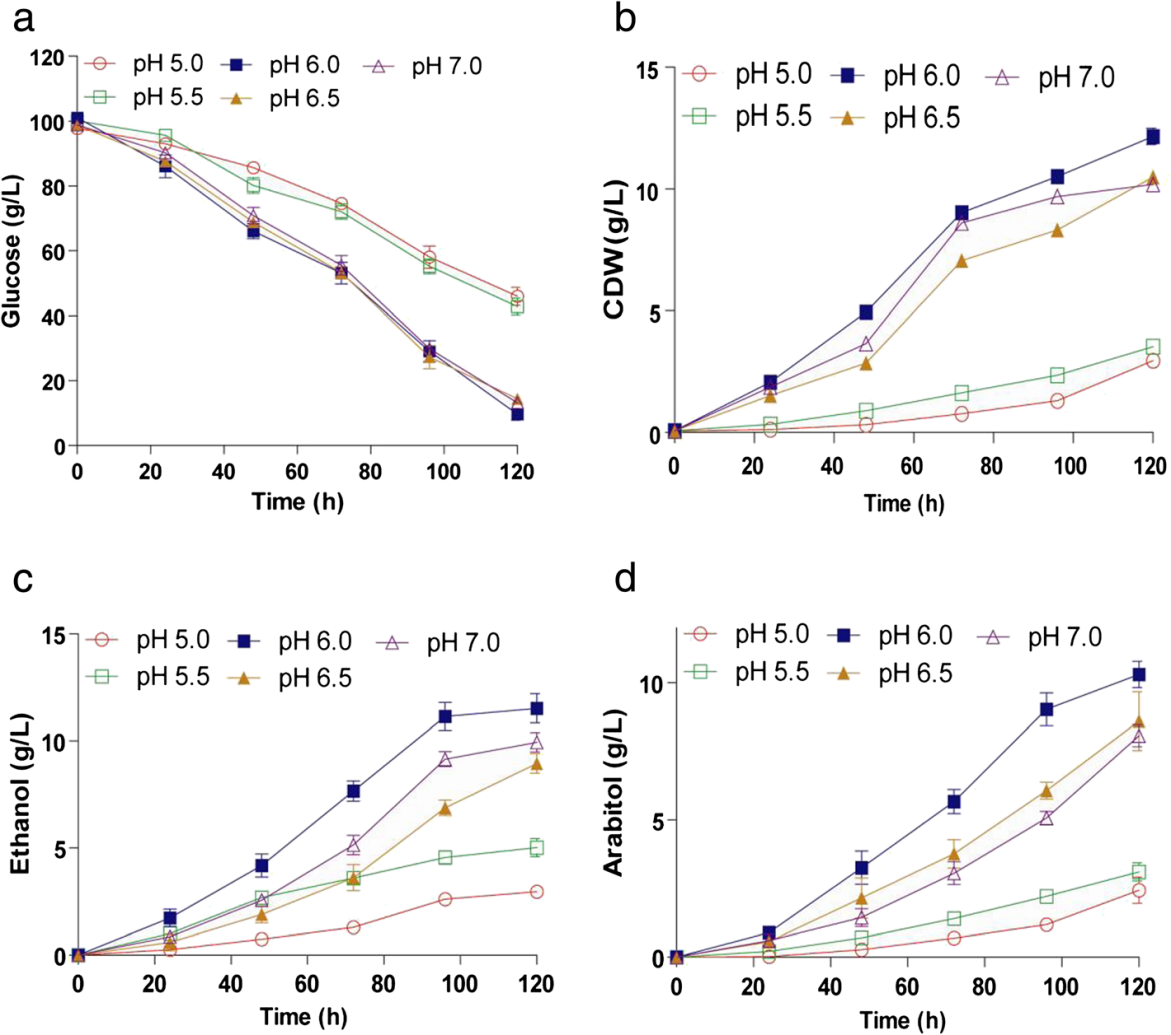
**Effect of initial pH on the production of ethanol and arabitol by *D. nepalensis*.** Time course of (**a**) substrate utilization, (**b**) growth and formation of (**c**) ethanol and (**d**) arabitol. The initial pH was varied in the range 5.0 to 7.0. Glucose was the carbon source and the cultivation conditions were same as mentioned in Figure 1. All experiments were performed three times and the values reported are mean with standard deviation.

*D. nepalensis* is known to survive over a wide pH range from 3.0 to 11.0 (Gummadi and Kumar 2006b Kumar and Gummadi 2011). In the interest of determining the optimum pH for growth and metabolite production, the initial pH of the culture medium was varied in the range 5.0 to 7.0. Both growth and substrate utilization were lower when the initial pH of the medium was 5.0 and 5.5 (Figure 2a, b). As seen in Figure 2a, pH 6.0-7.0 had negligible effect on the substrate utilization profile. However, metabolite production was strongly influenced by initial pH, as manifested by poor product yields at low pH. At pH 6.0, a maximum of 11.5 g/L ethanol and 10.4 g/L arabitol were produced (Figure 2c, d).

### Effect of nitrogen sources

The organism was grown in the presence of different sources of nitrogen like, ammonium sulphate, nitrates and nitrites along with yeast extract and its ability to produce ethanol and arabitol was studied. Among them, ammonium sulphate served as the best nitrogen source (data not shown), whereas, in the presence of nitrites and nitrates, the organism failed to metabolize glucose efficiently (data not shown). Yeast extract proved to be an integral source of amino acids and other vitamins for growth, without which, the organism had low efficiency for its metabolism.

**Figure 3:**
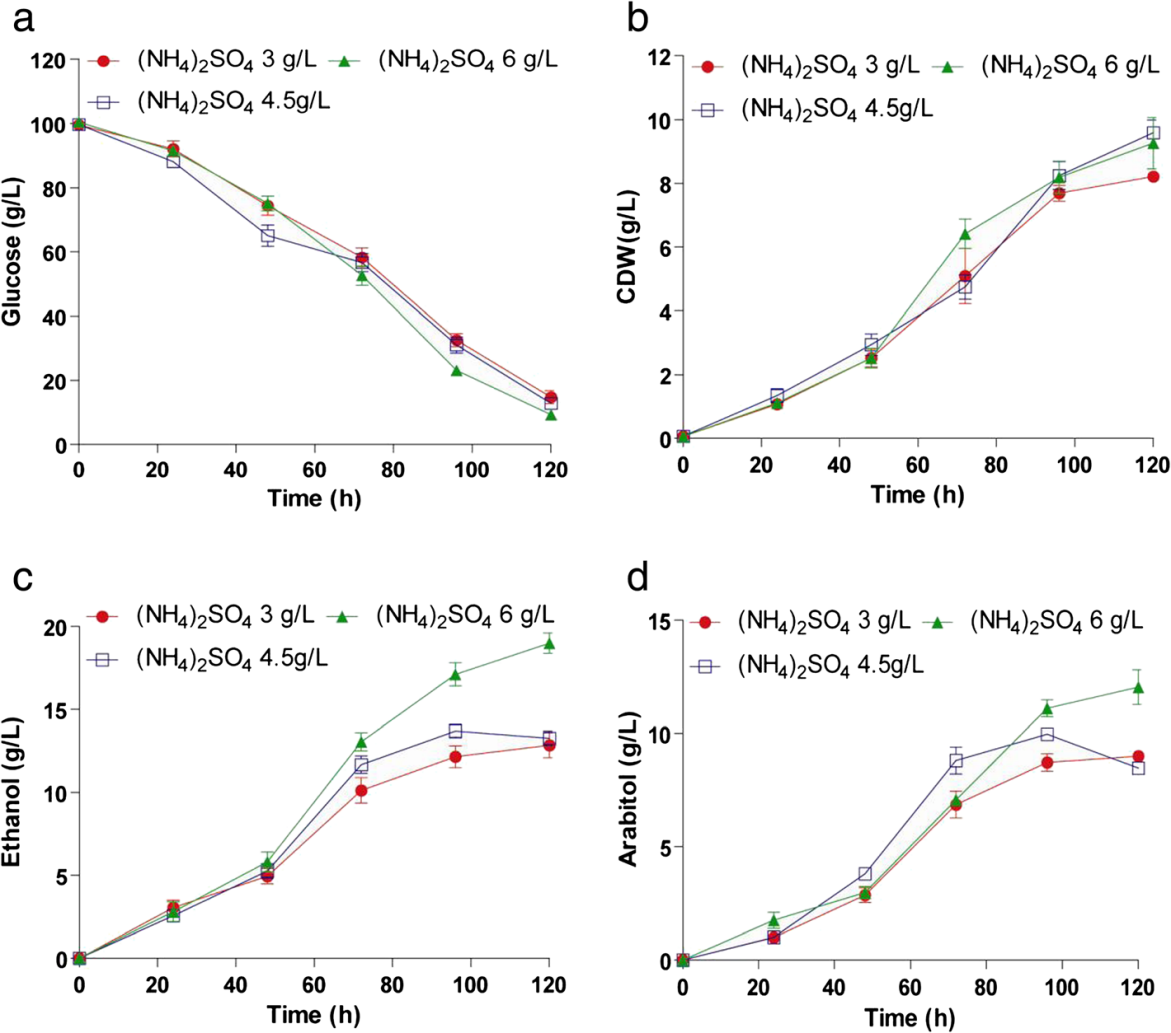
**Influence of varying concentration of nitrogen source on growth and product formation by *D. nepalensis *NCYC 3413.** Time course of (**a**) substrate utilization, (**b**) growth and formation of (**c**) ethanol and (**d**) arabitol. The medium was supplemented with different concentrations of ammonium sulphate (3–6 g/L). The initial pH was adjusted to 6.0 and the culture was incubated at 30°C and 180 rpm for 120 h. All experiments were performed three times and the values reported are mean with standard deviation.

**Figure 4:**
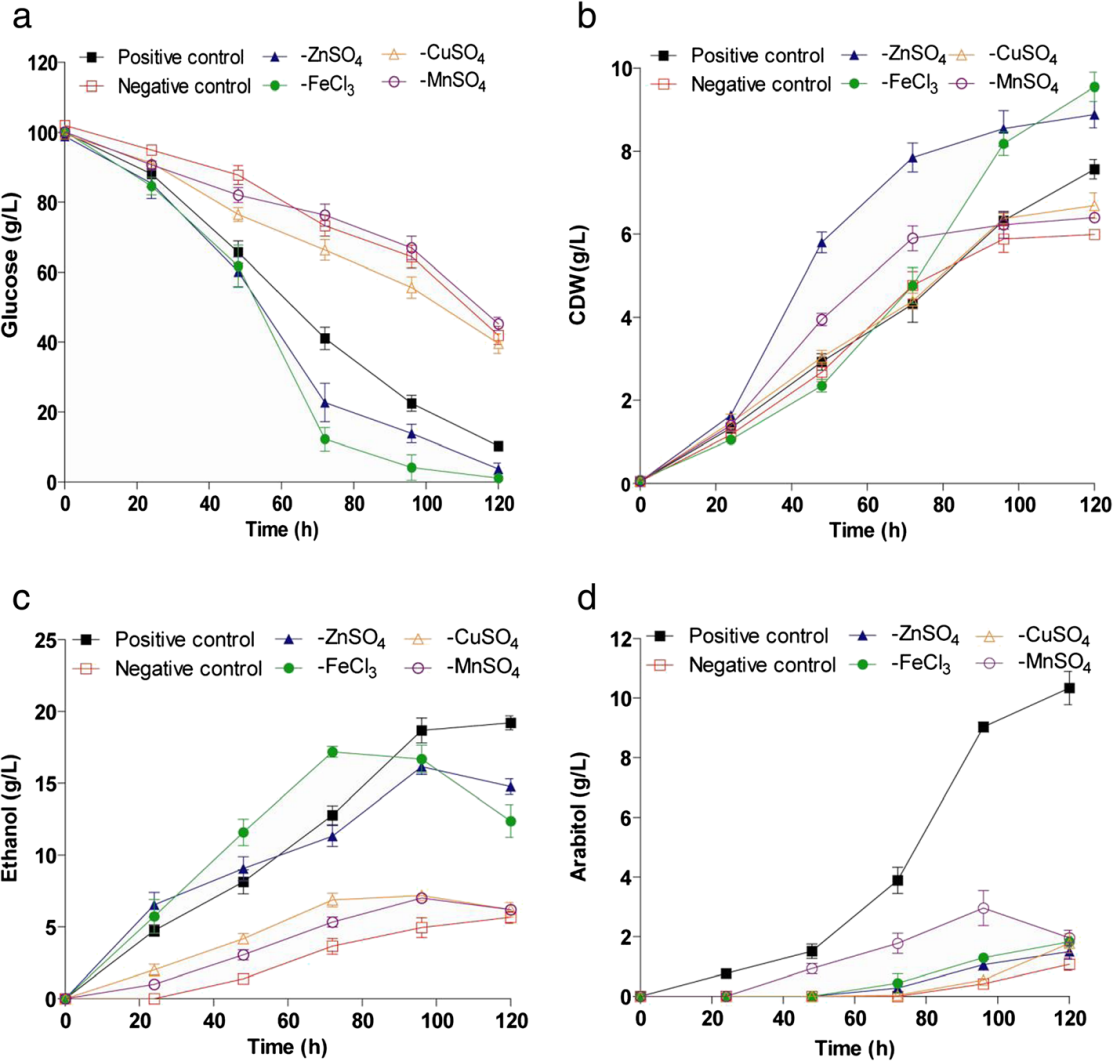
**Effect of trace elements on growth and product formation by *D. nepalensis *NCYC 3413.** The strain was grown at 30°C and 180 rpm in the presence or absence of trace elements and the cultivation conditions are: glucose 100 g/L; ammonium sulphate 6 g/L; pH 6.0. Time course of (**a**) substrate utilization, (**b**) growth and formation of (**c**) ethanol and (**d**) arabitol by *D. nepalensis*. All experiments were performed three times and the values reported are mean with standard deviation.

Further to determine the optimal concentration of ammonium sulphate, experiments were performed at 3, 4.5 and 6 g/L of ammonium sulphate. Biomass growth and substrate utilization were not considerably affected in this concentration range (Figure 3a, b). The organism produced 12 g/L of arabitol and 17 g/L ethanol in the presence of increased levels of ammonium sulphate (Figure 4c, d). The yield of arabitol and ethanol increased to 0.12 g/g and 0.21 g/g when 6g of (NH_4_)_2_SO_4_ was included in the medium (Table 1). Further increase to 7.5 and 9.0 g/L inhibited the growth and product formation (data not shown). Thus a balanced feed of D-glucose and ammonium sulphate, yeast extract was required for an increased efficiency in the rate of fermentation and growth.

### Effect of trace elements

To understand the influence of trace elements on yeast metabolism, the organism was cultivated in the presence and absence of four trace elements (Zn, Fe, Cu and Mn). The medium which is devoid of all the four trace elements served as a negative control, whereas positive control is inclusive of all of them. Each of these four trace elements was removed one at a time in order to define their individual effect. Figure 4 shows the effect of different metal ions on growth and product formation. It was found that the organism required Zn, Fe, Cu and Mn for growth as well as product formation. Growth was better in the absence of Zn and Fe, but very low in the case of negative control (Figure 4a). This can be directly correlated with the substrate utilization, which was faster and complete in the medium without Zn and Fe (Figure 4b). However, for the maximum production of ethanol and arabitol all the four trace elements are required (Figure 4c, d). The final concentration of ethanol and arabitol along with their respective yields was given in Table 1. The product yields were lower, when the trace elements concentration was maintained above and below the levels considered in this study (data not shown).

### Bioreactor studies

Under optimal conditions, as determined by the shake flask results, the production of ethanol and arabitol was also studied in 2.5 L bench top bioreactor, where, the effects of aeration and agitation rate on growth and metabolite production were appreciated.

#### Effect of aeration

**Figure 5:**
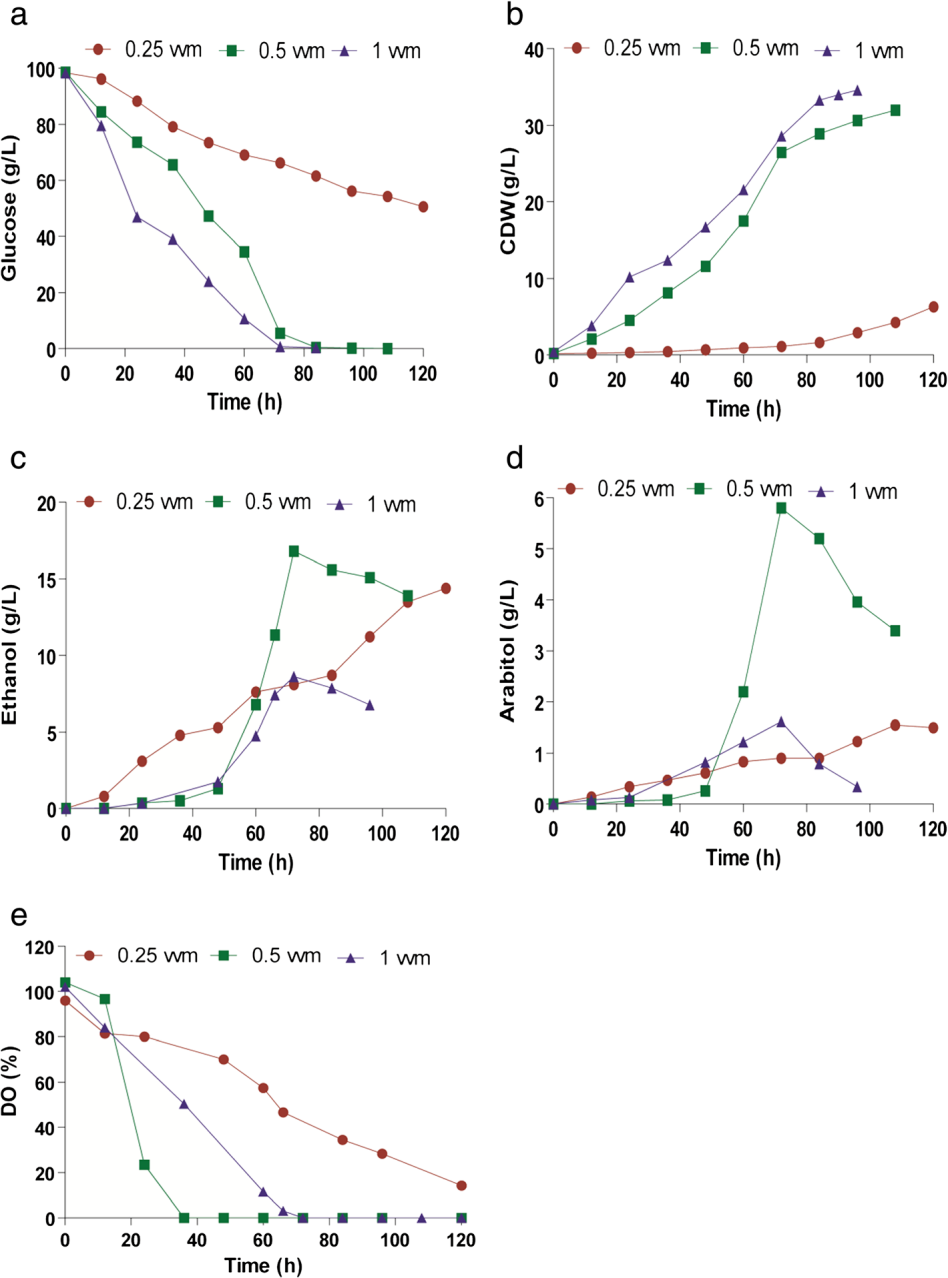
**Effect of aeration rate on the production of ethanol and arabitol by *D. nepalensis*.** Time course of (**a**) substrate utilization, (**b**) growth, formation of (**c**) ethanol and (**d**) arabitol and **e**) DO. The culture was performed in 2.5 L bioreactor containing 1.5 L production medium agitated at 700 rpm and different aeration levels (0.25-1 vvm).

Aeration and agitation are the prime factors considered during bioprocess scale-up. Initially, fermentation was performed at three different aeration rates such as 0.25, 0.5 and 1 vvm and a fixed agitation rate of 700 rpm. At 0.25 vvm, the growth was very low (4.5 g/L) which is attributable to poor substrate utilization (50%), as shown in Figure 5a. Complete utilization of glucose was attained in 96 h when the aeration rate was maintained at 0.5 vvm, with a high yield (0.33 g/g) of biomass (Figure 5a, b). Growth was favoured at high aeration rate (Figure 5b), but the metabolite production was maximum at 0.5 vvm (Figure 5c, d). The final concentrations of ethanol and arabitol at the end of fermentation were 14 g/L and 3.5 g/L respectively (Figure 5c and d). However, a maximum of 5.8 g/L of arabitol and 17 g/L of ethanol was produced by 84 h, a point where glucose was almost depleted and the products were utilized by the organism during later stages of fermentation. It should be noted that arabitol production was lower than shake flasks. The rate of substrate utilization was also significantly affected by varying aeration rate. At 1 vvm, the utilization was rapid and complete by 72 h, (Figure 5a) with most of the substrate being converted to biomass (36 g/L). The dissolved oxygen (DO) levels proportionately decreased with biomass growth (Figure 5e).

#### Effect of agitation speed

**Figure 6:**
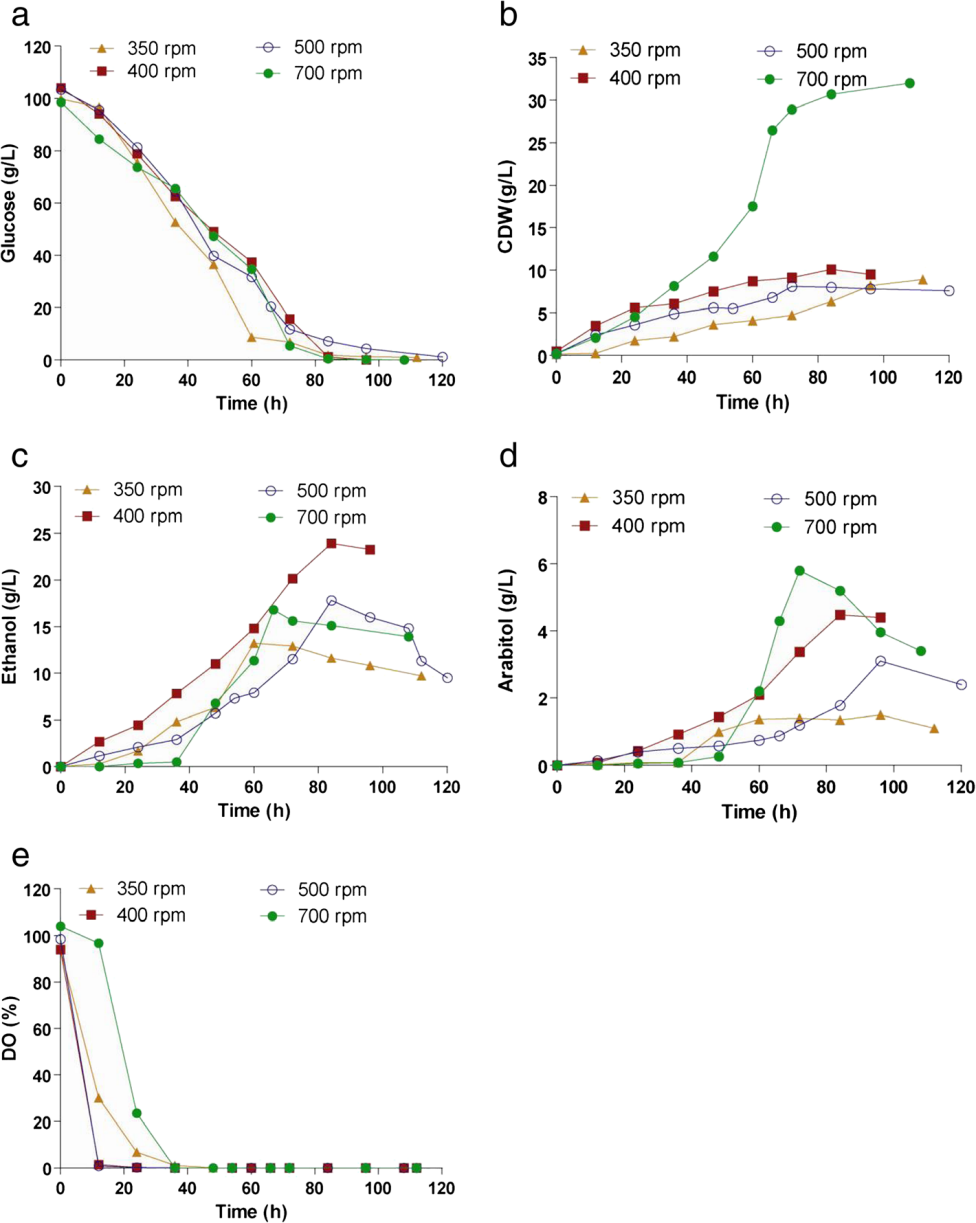
**Effect of agitation speed on the production of ethanol and arabitol.** Time course of (**a**) substrate utilization, (**b**) growth, formation of (**c**) ethanol and (**d**) arabitol and **e**) DO. Fermentation was done in 2.5 L bioreactor with a working volume of 1.5 L, agitated at different rates (350–700) and a fixed aeration rate of 0.5 vvm.

Based on the product yields achieved, an aeration level of 0.5 vvm was considered optimal for product formation. In order to determine optimum agitation rate, batch studies were done at 350, 400, 500 and 700 rpm while keeping the aeration rate constant at 0.5 vvm. The substrate utilization profile was almost similar under different agitation rates but maximum biomass was obtained at higher agitation rate of 700 rpm (Figure 6a, b). Decrease in agitation rate from 700 rpm to 400 rpm resulted in a maximum of 23.5 g/L of ethanol and 6.2 g/L of arabitol (Figure 6c, d). The yields of ethanol and arabitol with respect to substrate were 0.24 g/g and 0.06 g/g and the corresponding productivity values were 0.26 g/L/h and 0.07 g/L/h respectively (Table 2). Further decrease in agitation rate resulted in poor product yields. The dissolved oxygen concentration decreased with time, which is indicative of biomass growth (Figure 6e).

**Table 2:** Effect of different aeration and agitation rates on the yield of arabitol and ethanol

**Parameter**	**Substrate consumed (g/L)**	**Ethanol (g/L)**	**Arabitol (g/L)**	**Yield (g/g)**	
				**Ethanol**	**Arabitol**
**1. Aeration rate (vvm)**					
0.25	47.80	14.40	1.50	0.30	0.03
0.5	98.60	13.90	3.50	0.14	0.04
1	98.05	6.80	0.34	0.07	0.003
**2. Agitation(rpm)**					
350	98.80	13.00	1.40	0.13	0.01
400	104.10	23.90	4.50	0.23	0.04
500	102.20	3.10	17.80	0.03	0.17
700	98.60	13.90	0.04	0.14	0.04

#### Ethanol and arabitol production at high substrate concentration

**Figure 7:**
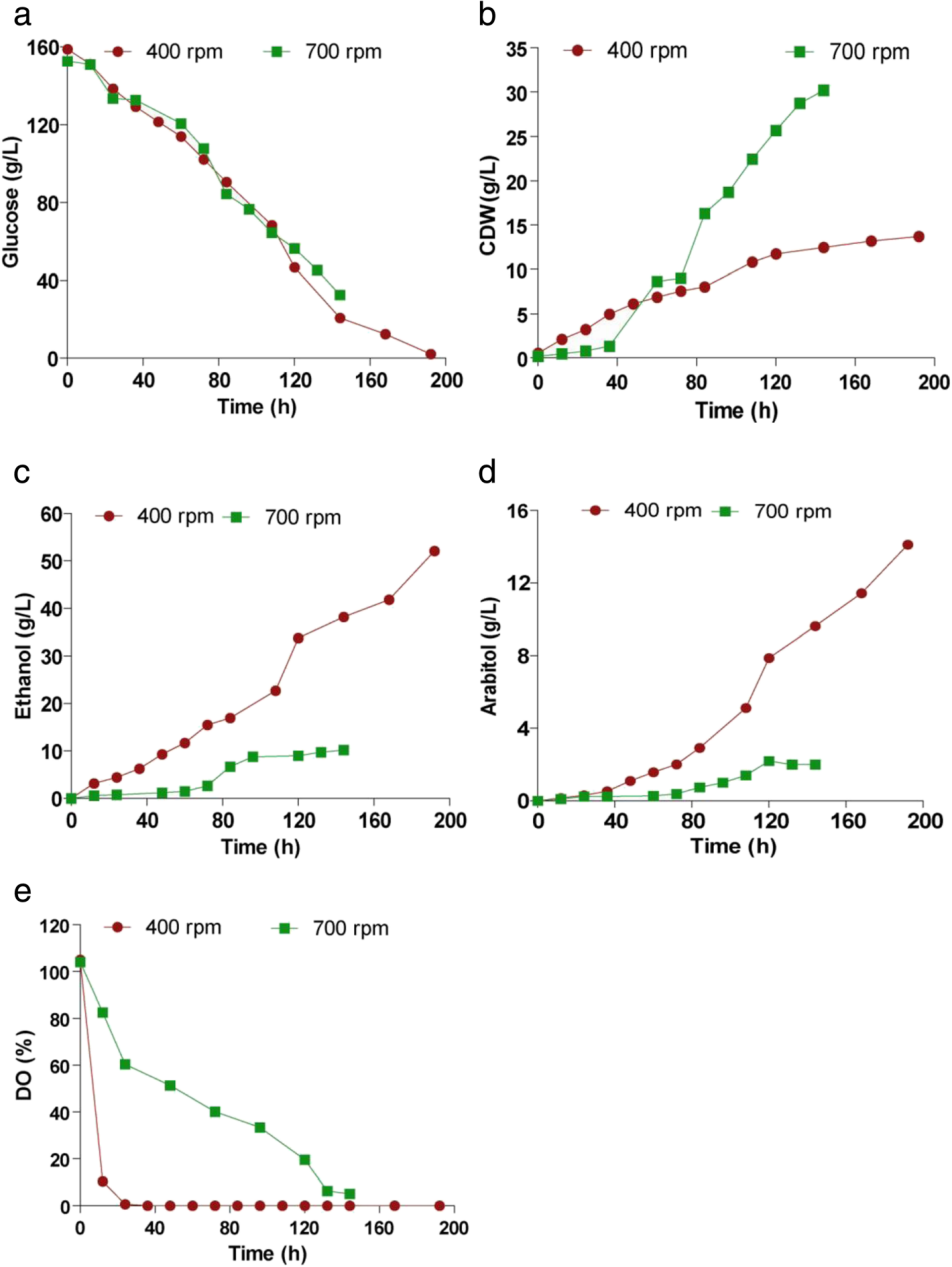
**Production of ethanol and arabitol at high initial substrate concentration.** Time course of (**a**) substrate utilization, (**b**) growth and formation of (**c**) ethanol and (**d**) arabitol and **e**) DO level during batch cultivation of D. nepalensis NCYC 3413 in 2.5 L bioreactor using a semi synthetic medium containing 150 g/L glucose, agitated at 400 & 700 rpm and aeration level was maintained at 0.5 vvm.

Since arabitol functions as an osmolyte in yeasts, it is obvious that arabitol production is greatly influenced by the sugar concentration in the medium. With this in view, we studied the effect of high initial substrate concentration on arabitol and ethanol production. The initial substrate concentration was increased to 150 g/L and fermentation was carried out at two different agitation speeds, 400 and 700 rpm, and an aeration rate of 0.5 vvm. The rate of substrate utilization and growth were slower during the initial phase of fermentation, when compared to other fermentations in this study, where 100 g/L of glucose was used. At reduced agitation speed of 400 rpm, much of the substrate consumed was converted to products instead of biomass (Figure 7a, b). Ethanol yield was remarkably enhanced to 0.34 g/g with a volumetric productivity of 0.28 g/L/h and the final concentration achieved was 52 g/L (Figure 7c, d). There was also an increase in the amount of arabitol obtained (14 g/L) which corresponds to a yield of 0.1 g/g and volumetric productivity of 0.08 g/L/h. When the broth was agitated at 700 rpm, a maximum of 30.2 g/L of biomass was obtained which gives a yield of 0.25 g/g. But the final concentrations of ethanol and arabitol were 9 g/L and 2 g/L respectively (Figure 7c, d). Unlike at 400 rpm, where the dissolved oxygen levels dropped rapidly, the rate of decrease was slow at 700 rpm. Higher K_L_a value (51.4 h^-1^), and the resultant high oxygen transfer rate, outpaced the rate of oxygen consumption, thus leading to a slow and gradual decrease in the DO level (Figure 7e). A comparison of fermentation parameters was given in Table 3.

**Table 3:** Kinetic parameters of ethanol and arabitol production at an initial substrate concentration of 150 g/L

**K**_**L**_**a (h**^**-1**^**)**	**Fermentation time (h)**	**Substrate consumed (g/L)**	**Ethanol (g/L)**	**Arabitol (g/L)**	**Y**_**P/S **_**(g/g)**		**Q**_**P **_(g/L/h)*	
					**Ethanol**	**Arabitol**	**Ethanol**	**Arabitol**
24.5	192	148.40	52.10	14.10	0.34	0.10	0.28	0.07
51.4	144	120.00	10.20	2.00	0.09	0.02	0.07	0.01

* Q_P_ is the ratio of final product concentration (g/L) to fermentation time (h).

## Discussion

It was previously reported that *D. nepalensis* produces arabitol and ethanol when grown on glucose (Kumar and Gummadi 2011). The present study focuses on understanding the effect of various process parameters on product formation, with subsequent development of suitable medium for the production of both ethanol and arabitol using glucose as carbon source. Studies on the production of arabitol and ethanol by *D. nepalensis* in the presence of different carbon substrates indicated that fructose and glucose were favourable for ethanol production. Shafaghat et al. (2009) have studied the effect of glucose, fructose and sucrose on the growth and ethanol productivity of *Saccharomyces cerevisiae* PTCC 24860 and observed similar results. The fermentation performance of the organism was found to be better when fructose was used as carbon source (Shafaghat et al. 2009). Arabinose, followed by glucose, were found to be more favourable carbon sources for arabitol production, the yield being greater (0.29 g/g) with arabinose. Similarly, Ahmed (2001) has shown that glucose was the suitable carbon source for bioconversion to arabitol by *Candida famata* R28. Nobre and Da Costa (1985) have also shown that arabitol production was better in medium containing arabinose and glucose. In contrast to the above observation, *Metschnikowia reukaufii* AJ14787 was unable to convert arabinose to arabitol (Nozaki et al. 2003). *D. nepalensis* was found to utilize glycerol only for the formation of biomass. On the contrary, Koganti et al. have reported the production of arabitol using glycerol as carbon source by *Debaryomyces hansenii* NRRL Y-7483 (Koganti et al. 2011). Based on experimental findings and comparison with literature suggest that there exists unique metabolism in *D. nepalensis* for concomitant production of ethanol and arabitol. Using glucose as carbon source and at pH 6.0 a final concentration of 12 g/L of ethanol was achieved. A similar pH range of 4–6 was shown to be optimal for ethanol production by *Kluyveromyces marxianus* (Bajpai and Margaritis 1987) and *Zymomonas mobilis* (Lawford et al. 1988). Arabitol production was also maximum at pH 6.0 (Saha et al. 2007; Zhu et al. 2010).

An increase in the concentration of nitrogen in the medium has a positive effect on ethanol and arabitol production. In the present study, the yield of both ethanol and arabitol increased with increasing nitrogen concentration (or decreasing C/N ratio). This could be due to efficient utilization of glucose for product formation. But when the nitrogen concentration was further increased to 9 g/L, ethanol yield was reduced drastically to 0.09 g/g and there was a significant reduction in the percentage of glucose consumed (40% of the total glucose). Similar observation on the effect of nitrogen source was made by Abd-Aziz et al. (2001) on ethanol production by recombinant *Saccharomyces cerevisiae* YKU 131. It was also reported that decreasing C/N ratio resulted in poor ethanol yield (Manikandan and Viruthagiri 2010). Excessive nitrogen concentration was also shown to be unfavourable for arabitol production by *Kodamae ohmeri* (Zhu et al. 2010)*.*

Trace elements such as Zinc, Mn, Cu, & Fe are involved in yeast metabolism as cofactors for enzymes and as components of respiratory pathways. Hence, it is obvious that, in the absence of these trace factors, metabolism would be impaired. Supplementing 0.5 mM Cu to the growth medium was found to enhance ethanol production by *Saccharomyces* (Azenha et al. 2000; McHargue and Calfee 1931). Similarly Zn, Fe and Mn were also shown to effect metabolism and by product formation in many yeasts and bacteria (Azenha et al. 2000; McHargue and Calfee 1931; Reeslev and Jensen 1995; Vesna et al. 2004; Fitzpatrick et al. 2001).

After selecting the appropriate ranges of medium components, the production of ethanol and arabitol was scaled up to 2.5 L bioreactor and the optimum aeration/agitation conditions were established. Oxygen is one of the important substrates for microbial metabolism. There were many reports indicating the necessity of adequate supply of oxygen for polyol production (Petrson et al. 1958; Hajny 1964; Escalante et al. 1990; Saha et al. 2007; Toyoda and Ohtaguchi 2011). In the present study, production of ethanol and arabitol was found to be maximum at lower rates of aeration and agitation. Drastic reduction of product yields and high biomass were observed when aeration was increased to 1 vvm. This could be due to the Pasteur effect, as indicated by Petrson et al. (1958) where higher levels of oxygen reduce the rate of fermentation. Several reports indicated that low levels of aeration and agitation are optimal for arabitol production (Petrson et al. 1958; Fonseca et al. 2007; Saha et al. 2007). Saha et al. (2007) studied the production of arabitol by *Z. rouxii* under different agitation rates and found that low agitation rates in the range 350–450 rpm were most conducive. But, in contrast to the above observation, Escalante et al. (1990) reported an increase in arabitol yield with increasing aeration and agitation speed. The partial anaerobic environment created by reduced agitation speed and low aeration rates resulted in maximum ethanol yield. When the initial substrate concentration was increased, the production of both ethanol and arabitol also increased. The increase in arabitol yield can be attributed to the osmotic stress caused by high sugar concentration. This is in agreement with earlier published report where *D. nepalensis* produced arabitol as a compatible solute along with glycerol to overcome hyperosmotic stress (Kumar and Gummadi 2011). This result also corresponds to the observations made by Morant et al. that arabitol production increased when *Z. rouxii* was cultivated in the presence of high glucose concentrations (Morant and Witter 1979). Similarly, Brown (1974) reported that the amount of arabitol produced by *Saccharomyces rouxii* was influenced by the solute concentration of the medium in which the organism was cultivated. But, in the case of *Hansenula polymorpha*, there was a reduction in arabitol production with increasing osmotic pressure (Escalante et al. 1990).

In this work, optimal conditions for the production of arabitol and ethanol were determined. Under these conditions, the final product concentrations were significant for industrial application. It can be concluded that by manipulation of various fermentation parameters, it is possible to favour the production of desired metabolite. The ability of *D.nepalensis* NCYC 3413 to ferment different sugars was analyzed. The organism utilises glucose substantially to produce bioethanol and arabitol. Since it shows utilization of simple sugars efficiently, exploitation of this organism in conversion of raw substrate rich in simple sugars needs to be explored.

## Competing interests

The authors declare that they have no competing interests.

## Authors’ contributions

SNG, HK and SNM designed the experiment, analyzed the data and drafted the manuscript. HK and SNM performed experiments and collected data. All authors read and approved the final manuscript.
